# Communicating Science in the Digital and Social Media Ecosystem: Scoping Review and Typology of Strategies Used by Health Scientists

**DOI:** 10.2196/14447

**Published:** 2019-09-03

**Authors:** Guillaume Fontaine, Marc-André Maheu-Cadotte, Andréane Lavallée, Tanya Mailhot, Geneviève Rouleau, Julien Bouix-Picasso, Anne Bourbonnais

**Affiliations:** 1 Faculty of Nursing Université de Montréal Montréal, QC Canada; 2 Research Center Montreal Heart Institute Montréal, QC Canada; 3 Research Center Université de Montréal Hospital Center Montréal, QC Canada; 4 Research Center CHU Sainte-Justine Montréal, QC Canada; 5 Department of Pharmacy and Health Systems Sciences Bouvé College of Health Sciences Northeastern University Boston, MA United States; 6 Faculty of Nursing Université Laval Québec City, QC Canada; 7 Health Education and Practice Laboratory The Faculty of Health, Medicine and Human Biology Université Paris 13 Nord Paris France; 8 French Military Health Service Academy Department for Non-Medical Personnel Education École du Val-de-Grâce Paris France; 9 Research Center Institut Universitaire de Gériatrie de Montréal Montréal, QC Canada

**Keywords:** health communication, public health, social media, internet, patient participation

## Abstract

**Background:**

The public’s understanding of science can be influential in a wide range of areas related to public health, including policy making and self-care. Through the digital and social media ecosystem, health scientists play a growing role in public science communication (SC).

**Objective:**

This review aimed to (1) synthesize the literature on SC initiated by health scientists targeting the public in the digital and social media ecosystem and (2) describe the SC strategies and communication channels used.

**Methods:**

This scoping review was based on the Joanna Briggs Institute Methodological Framework. A systematic search was performed in 6 databases (January 2000 to April 2018). Title and abstract screening, full-text review, data charting, and critical appraisal were performed independently by two review authors. Data regarding included studies and communication channels were synthesized descriptively. A typology of SC strategies was developed using a qualitative and inductive method of data synthesis.

**Results:**

Among 960 unique publications identified, 18 met inclusion criteria. A third of publications scored good quality (6/18, 33%), half scored moderate quality (9/18, 50%), and less than a fifth scored low quality (3/18, 16%). Overall, 75 SC strategies used by health scientists were identified. These were grouped into 9 types: content, credibility, engagement, intention, linguistics, planification, presentation, social exchange, and statistics. A total of 5 types of communication channels were identified: social networking platforms (eg, Twitter), content-sharing platforms (eg, YouTube), digital research communities (eg, ResearchGate), personal blogs and websites (eg, WordPress), and social news aggregation and discussion platforms (eg, Reddit).

**Conclusions:**

Evidence suggests that multiple types of SC strategies and communication channels are used by health scientists concurrently. Few empirical studies have been conducted on SC by health scientists in the digital and social media ecosystem. Future studies should examine the appropriateness and effectiveness of SC strategies for improving public health–related outcomes and identify the barriers, facilitators, and ethical considerations inherent to the involvement of health scientists in the digital and social media ecosystem.

## Introduction

### Background

The public’s understanding of science can be influential in a wide range of areas related to public health, including policy making and self-care [1,2]. Although the public uses digital and social media primarily to network and nurture social connections, individuals are frequently exposed to various types of information related to, for instance, politics and health [3]. Thus, some organizations suggest scientists should use their expertise and influence to communicate science in the digital and social media ecosystem to change people’s health-related attitudes, behaviors, and policy preferences [4]. However, this task is complexified by the prevalence of misinformation on social media and the ease with which this content can be propagated to audiences targeted by increasingly sophisticated algorithms [3].

Science communication (SC), in the context of health sciences, is a process of knowledge exchange about health-related scientific information or viewpoints [4]. SC falls within the broader domain of the mass communication of scientific and biomedical evidence including, among other things, health communication interventions, numeracy, and health literacy [4-7]. The process of SC encompasses multiple stakeholders, integrates strategies and goals operationalized through various communication channels, and involves numerous audiences. In recent years, a new dimension emerged in the SC literature: the direct relationship between scientists and laypeople (hereafter *the public*) [2,8,9].

The internet is the primary source of information for almost 70% of the public looking for information about scientific topics [10]. Through the growth of digital and social media, new, direct, and powerful communication channels between scientists and the public were enabled, allowing for the disintermediation of SC [10]. Disintermediation refers to the public’s direct access to scientific information from scientists through the social and digital media ecosystem, a process that would otherwise require a human mediator such as a journalist [11,12]. Health scientists are thus increasingly expected to perform SC, often as an institutional requirement, or to integrate SC in their research program [10]. However, SC can be a complex endeavor as it is tailored to the motives, time commitments, and resources of each scientist and the information that they intend to communicate [4]. In light of this, several health scientists seek information and resources on how to communicate science.

Indeed, although health scientists are used to communicating science to their peers, they might be less familiar with the use of appropriate SC strategies and communication channels with the public [13]. In this context, questions arise regarding how health scientists should effectively engage the public in SC through the social and digital media ecosystem. Effective public communication of science requires specialized knowledge and skills. Multiple factors contribute to the complexity of SC. First, scientific information is complex, and people perceive and process this information in different ways. Thus, different SC strategies may be used for audiences with variable levels of science literacy. Second, the process of SC involves uncertainty, “either in the science itself or its implications or as a result of various communicators conveying different, and sometimes contradictory, messages” [4]. Health scientists must consider the appropriate strategies to convey this uncertainty during the process of SC. Third, social influences play an important part in SC, a phenomenon that may be exacerbated when science is communicated in the digital and social media ecosystem. Indeed, social networks affect people’s beliefs, attitudes, and behaviors [4].

### Objectives

To our knowledge, no review has previously attempted to identify the nature and extent of the evidence regarding SC initiated by health scientists in the digital and social media ecosystem. Thus, the primary objective of this scoping review was to describe the nature and the extent of the literature regarding SC initiated by health scientists and targeting the public in the digital and social media ecosystem. The secondary objective of this scoping review was to describe the SC strategies and communication channels used by health scientists in this context.

## Methods

### Methodological Framework

We planned and conducted this scoping review following the Joanna Briggs Institute Methodological Framework [14]. Scoping reviews aim to “examine the extent (that is, size), range (variety), and nature (characteristics) of the evidence on a topic or question” [15]. This scoping review is reported according to the Preferred Reporting Items for Systematic Reviews and Meta-Analyses (PRISMA) extension for scoping reviews [15].

### Protocol and Registration

We previously published the protocol of this scoping review [12]. There was no registration of the protocol as scoping reviews are not eligible according to the International Prospective Register of Systematic Reviews. In this study, we present an abridged version of the methods employed.

### Eligibility Criteria

We included any type of literature (eg, gray literature and original research paper) published in English or in French, between 2000 and 2018, if the inclusion criteria were met regarding population, concept, and context.

#### Population

We considered the literature reporting SC strategies involving disintermediation used by health scientists with the public. Regarding health scientists, we included literature about scientists in all health disciplines, as defined by the classification of the World Health Organization (eg, medicine, dentistry, pharmacy, and psychology) [16]. Regarding the public, we included literature involving the public at large (ie, laypeople) or specific sociodemographic groups (eg, teenagers, young adults, and women). However, we excluded the literature involving specifically patients and students as other fields of study relate directly to these populations (ie, patient education and health sciences education).

#### Concept

We included the literature that described a process of SC involving health scientists and the public operationalized through communication channels in the digital and social media ecosystem.

A recent typology summarized 4 types of SC [17]. Type 1 (professional SC) refers to knowledge exchanged among scientists. Type 2 (deficit SC) refers to knowledge unidirectionally exchanged from scientists to the public. Type 3 (consultative SC) refers to knowledge exchanged bidirectionally and iteratively between scientists and the public. Type 4 (deliberative SC) is defined as “knowledge exchanged in a democratic and deliberative manner in which the principal actors have equal standing, and scientific knowledge and local knowledge are mutually respected” [17]. Type 1 is thus beyond the scope of this review as it does not involve the public. We adopted a broad definition of SC that encompasses all types of SC involving scientists and the public (types 2, 3, and 4). We defined SC as an interactive process of knowledge exchange between scientists and the public using SC strategies through various communication channels.

Furthermore, we defined a SC strategy as any plan or action adopted by scientists (eg, using humor, disseminating research findings using images, and telling a story) to communicate science.

#### Context

We included sources reporting SC strategies used by scientists with the public in the digital and social media ecosystem about any topic or area related to clinical aspects of health.

### Information Sources and Search

We drafted the search strategy with an experienced librarian. We first developed the search strategy for PubMed (see Multimedia Appendix 1) and then translated it for other databases. The search strategy used a combination of 3 concepts: (1) scientists and the public; (2) health SC; and (3) disintermediation, which refers to the communication channels in the digital and social media ecosystem. We searched 6 bibliographical databases from January 2000 up until April 2018: Cumulative Index to Nursing and Allied Health Literature, via EBSCOhost; Excerpta Medical Database, via Ovid; International Bibliography of the Social Sciences, via ProQuest; PubMed, via the National Center for Biotechnology Information; Sociological Abstracts, via ProQuest; and Web of Science—Science Citation Index and Social Sciences Citation Index, via the Institute for Scientific Information—Thomson Scientific. We also searched relevant gray literature sources, trial registries, and journals. Finally, we screened the reference lists of included records to identify additional records. We exported search results into EndNote V8.0 (Clarivate Analytics), and we removed duplicates.

### Selection of Sources of Evidence

We worked independently and in duplicate (GF and AL or TM) to screen titles and abstracts and resolved disagreements through consensus. We then performed the full-text review of potentially eligible articles using the same method (GF and GR or MAMC or JBP).

### Data Charting Process

A data charting form was developed specifically for this review. A total of 6 review authors were involved in the data charting process (GF, AL, MAMC, TM, GR, and JBP). Thus, to ensure accuracy and prevent inconsistencies, we completed a pretest of the data charting process, after which adjustments were made to the data charting form. Clarifications regarding the type of publication and how to assess the quality of the articles were the main changes made. Working in teams of 2, reviewers independently charted the data, discussed the results, and completed a “consensus” form for each included publication.

### Data Items

We extracted the following data items:

- Article characteristics (eg, year of publication, first author’s academic discipline, country of origin, publication type, and aim)
- SC-related items (eg, SC definition, SC type, SC theoretical framework, SC goal, SC context, SC strategies used or described, SC communication channels used or described, SC plan development process, and SC delivery)
- Study methods, if applicable (eg, study design, population, sample size, data collection and analysis method, and article limits)
- Key results related to SC

### Critical Appraisal of Individual Sources of Evidence

Although critical appraisal of the included publications was not originally planned [12], review authors consensually decided to add this step to further qualify the literature. We critically appraised empirical works, which are original research articles and literature reviews, based on general guidance (eg, coherence between the problem described and the methods retained, adequate sample in terms of participants or the literature selected, and rigor of the data collection or extraction process). To critically appraise nonempirical types of articles (eg, editorials and viewpoints), we retained the Joanna Briggs Institute Critical Appraisal Checklist for Text and Opinion Papers [18].

### Synthesis of Results

We first synthesized data regarding the nature and the extent of the literature regarding SC initiated by health scientists in the digital and social media ecosystem in a table format. Then, we synthesized narratively data regarding SC-related data items (eg, communication channels).

To develop a typology of SC strategies, we used a qualitative and inductive method of data synthesis, with a constant comparison approach [19]. This method of data synthesis allowed the types of SC strategies to emerge from the available data and not from prespecified categories. First, we listed in a single file all SC strategies identified in included publications. Second, 2 reviewers (GF and MAMC) consensually attached a provisional label to each SC strategy to identify its type or, in other words, its focus. For example, the strategy “Developing a plan for engaging the targeted audience” was labeled “Planification,” and the strategy “Use numbers instead of words when possible” was labeled “Linguistics.” Third, all provisional types of SC strategies identified during this first round were compared and contrasted to identify similar ones and regroup those with similar focus. For example, the provisional types of SC strategies labeled “Structure” and “Presentation” were grouped together under the latter. Indeed, all SC strategies dealing with the structure of the information to be disseminated were closely related to the visual presentation of the information. Finally, the proposed typology was sent to a third reviewer experienced in qualitative data synthesis (AB) not involved yet in the previous steps for validation.

## Results

### Selection of Sources of Evidence

**Figure 1:**
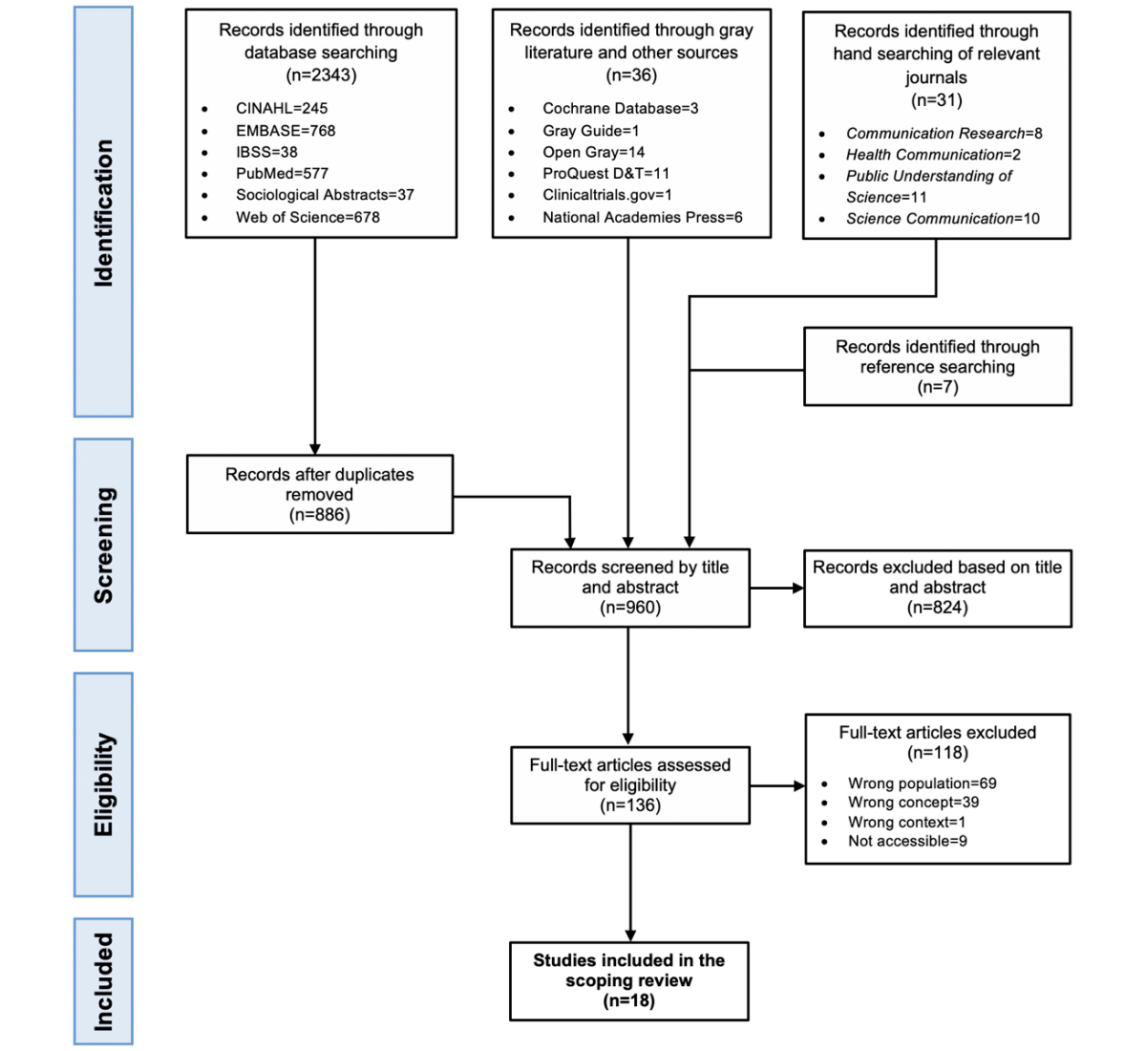
Study flow diagram. CINAHL: Cumulative Index to Nursing and Allied Health Literature; EMBASE: Excerpta Medical Database; IBSS: International Bibliography of the Social Sciences; D&T: Dissertations and Theses.

From a pool of 960 unique publications, we assessed 136 full texts for eligibility, and we included 18 publications that described a process of SC operationalized in the digital and social media ecosystem involving health scientists and the public, as illustrated in the PRISMA study flow diagram [20] in Figure 1.

### Characteristics of Sources of Evidence

The characteristics of included sources of evidence are reported in Table 1. Overall, publications originated from a wide variety of disciplines. Types of publications were varied; we included primary research articles (4/18, 21%), conference proceedings (4/18, 21%), primary research abstracts (2/18, 11%), reports (2/18, 11%), discussion papers (2/18, 11%), an editorial (1/18, 5%), a review (1/18, 5%), a scientific poster (1/18, 5%), and a quality improvement paper (1/18, 5%). The aims of publications were also varied: 7 discussed the benefits and implications of using social media for communicating science to the public [21-26]; 3 studies aimed to develop, test, and disseminate lay summaries of evidence [27-29]; 2 publications aimed to discuss various prospects, priorities, and strategies for improving SC with the public [4,10]; 1 publication summarized articles regarding prospects for SC in the Web 2.0. era [30]; 1 publication presented international SC experiences [31]; 1 publication aimed to provide guidance on how to identify the public’s information needs and conduct deliberative SC [32]; 1 publication advocated for adopting a community-partnered participatory research approach to SC [33]; 1 publication aimed to develop and evaluate a Web-based research advisory community [34]; and 1 publication aimed to explore the concept of science literacy in relation to SC [35]. We also identified the type of SC described in each publication according to the classification of Palmer and Schibeci [17]. A total of 6 publications (6/16, 38%) described *deficit SC* (type 2), 4 publications (4/16, 25%) described *consultative SC* (type 3), and 6 publications (6/16, 38%) described *deliberative SC* (type 4). We were unable to determinate the type of SC for 2 publications. Only 2 publications (2/18, 16%) referred to a theoretical framework. Archibald and Clark [21] suggested using the Diffusion of Innovations Theory [36] to promote understanding of how scientists use social media and offer insight for increasing its use in academic settings by considering the 5 characteristics of innovation: relative advantage, compatibility, complexity, trialability, and observability. Russell and Sprung [34] based the development of their Web-based research advisory community on the model of knowledge translation proposed by Holmes and Scarrow [37].

**Table 1:** Characteristics of sources of evidence.

First author or institution, year; country	Academic background of the first author	Publication type	Publication aim	Type of SC^a^
Archibald, 2014 [21]; Canada	Nursing	Editorial	To describe how Twitter and the Diffusion of Innovation Theory can help uptake of nursing research	Deficit SC (type 2)
Barnfield, 2017 [27]; United Kingdom	Biomedical sciences	Primary research article	To select a series of studies from the Newcastle Cognitive Function after Stroke cohort and create lay summaries comprehensible and accessible to the public	Consultative SC (type 3)
Bin, 2012 [22]; Australia	Epidemiology	Primary research abstract	To explore the relative costs and benefits of communicating sleep research using social media to a lay audience	NR^b^
Bodison, 2015 [33]; United States	Occupational science	Conference proceedings	To advocate for the use of CPPR^c^ practices in dissemination, implementation, and improvement science and to offer insight about barriers and solutions to CPPR success in a large, urban community	Deliberative SC (type 4)
Breland, 2017 [23]; United States	Implementation science and psychology	Discussion paper	To describe 5 benefits for public health scientists of disseminating their work via social media	Deficit SC (type 2)
Finch, 2012 [24]; Australia	Sports medicine	Primary research abstract	To describe experiences over the past months of using Twitter, LinkedIn, and blogging and summarize some of the approaches that can be used with these social media tools and show how to encourage interaction among scientists, practitioners, and general public	NR
Fordis, 2011 [30]; United States	Medicine	Conference proceedings	To summarize key articles regarding prospects for Web 2.0 technologies for engagement, communication, and dissemination in the era of patient-centered outcomes research	Deliberative SC (type 4)
Glenton, 2010 [28]; Norway	Implementation science	Primary research article	To develop and test a summary of evidence that a consumer audience would understand and obtain feedback about different versions of a format for a Plain Language Summary of a Cochrane Systematic Review	Deficit SC (type 2)
Lafferty, 2015 [25]; United Kingdom	Medical education	Review	To review some of the emerging evidence and commentaries on the adoption and role of social media in research, which may inform their further application in medical and health care research	Consultative SC (type 3)
Miranda, 2014 [31]; United Kingdom	Hygiene and tropical medicine	Scientific poster	To present dissemination experiences from the Artemisinin-based Combination Therapy Consortium, a global research partnership with 25 projects in 10 countries aiming to improve malaria drug delivery and use	Deficit SC (type 2)
National Academy of Sciences, Engineering and Medicine, 2013 [32]; United States	Multiple disciplines	Conference proceedings	To describe colloquiums that brought together leading social, behavioral, and decision scientists to familiarize one another, other scientists, and communication practitioners with current research that can improve the communication of science to lay audiences	Deliberative SC (type 4)
National Academy of Sciences, Engineering and Medicine, 2016 [10]; United States	Multiple disciplines	Conference proceedings	To summarize the workshop’s presentations and discussions, and it recounts what workshop participants identified as key lessons, practical strategies, and the needs and opportunities for applying the principles of health literacy to the precision medicine	Deliberative SC (type 4)
National Academy of Sciences, Engineering and Medicine, 2017 [4]; United States	Multiple disciplines	Report	To offer a research agenda for science communicators and researchers seeking to apply this research and fill gaps in knowledge about how to communicate effectively about science, with a particular focus on issues that are contentious in the public sphere	Deliberative SC (type 4)
Rowe, 2017 [26]; United States	Nutrition	Discussion paper	To offer some insight into the effect that rapidly evolving social and other digital media may have on the various perceptual influences on SC in the field of nutrition	Deficit SC (type 2)
Russell, 2016 [34]; Canada	Kinesiology and pediatrics	Quality improvement project paper	To describe the development and evaluation of a Web-based research advisory community, hosted on Facebook and connecting a diverse group of parents of special needs children with researchers at CanChild Centre for Childhood Disability Research	Deliberative SC (type 4)
Santesso, 2015 [29]; Canada	Nutrition	Primary research article	To compare a new format of a patient summary of evidence from a systematic review with the current narrative format and evaluate if it improves understanding, accessibility of the information, and whether it is preferred over other versions by patients and the public	Deficit SC (type 2)
Snow, 2016 [35]; United States	Psychology and education	Report	To consider how the definition of science literacy has expanded and shifted over time to accommodate changing ideas about science	Consultative SC (type 3)
Tunnecliff, 2015 [38]; Australia	Physiotherapy	Primary research article	To explore health scientists’ and clinicians’ current use of social media and their beliefs and attitudes toward the use of social media for communicating research evidence	Consultative SC (type 3)

^a^SC: science communication.

^b^Not reported.

^c^CPPR: community-partnered participatory research.

### Critical Appraisal of Individual Sources of Evidence

Results of the critical appraisal of individual sources of evidence are presented in Table 2. Overall, 6 publications scored good quality (6/18, 33%), 9 scored moderate quality (9/18, 50%), and 3 scored low quality (3/18, 16%). Regarding the quality of empirical studies, 1 criterion in particular was unclear: if the sample size used was adequate to reach the study goal. Regarding the quality of other types of publications, 2 criteria were often unclear or not properly reported. First, there was often no reference during the discussion to the extant literature in the field of SC to contrast or support the author’s opinion. Second, incongruences with the cited sources were often not logically defended by the author, undermining the credibility of the opinion.

### Results of Individual Sources of Evidence

We identified 9 types of SC strategies used by health scientists with the public in the digital and social media ecosystem: content, credibility, engagement, intention, linguistics, planification, presentation, social exchange, and statistics. Definitions and examples of each type of SC strategy are presented in Table 3. Results suggest health scientists use a wide variety of SC strategies, with different purposes. Some strategies are related to the content and credibility of the message, some are related to linguistics and statistics to improve the public’s understanding of science, whereas others aim to increase engagement and social exchange related to science in the social and digital media ecosystem.

**Table 2:** Quality of included sources of evidence.

First author or institution, year	Empirical studies and literature reviews				Other types of publications						Overall quality
	Coherence between problem, purpose, methods, and results?	Research process meets scientificity criteria?	Sample adequate to reach goal?	Data collection and analysis rigorous?	Source of the opinion clearly identified?	Source of the opinion has standing in the field of expertise?	Interests of the relevant population the central focus?	Stated position the result of an analytical process?	Reference to the extant literature?	Any incongruence with the sources logically defended?	
Archibald, 2014 [21]	—^a^	—	—	—	Yes	Unclear	Yes	Yes	Yes	Unclear	Good
Barnfield, 2017 [27]	Yes	Yes	Unclear	Unclear	—	—	—	—	—	—	Moderate
Bin, 2012^b^ [22]	Yes	Yes	Unclear	Yes	—	—	—	—	—	—	Moderate
Bodison, 2015 [33]	—	—	—	—	Yes	Unclear	Yes	Yes	Unclear	Unclear	Moderate
Breland, 2017 [23]	—	—	—	—	Yes	Unclear	Yes	Unclear	No	No	Moderate
Finch, 2012^b^ [24]	—	—	—	—	Yes	Unclear	Unclear	Unclear	No	No	Low
Fordis, 2011 [30]	—	—	—	—	Yes	Yes	Yes	Yes	Unclear	No	Good
Glenton, 2010 [28]	Yes	Unclear	Unclear	Unclear	—	—	—	—	—	—	Moderate
Lafferty, 2015 [25]	Yes	Unclear	Unclear	Unclear	—	—	—	—	—	—	Low
Miranda, 2014 [31]	—	—	—	—	Yes	Unclear	No	No	Unclear	No	Low
NASEM, 2013 [32]	—	—	—	—	Yes	Yes	Unclear	Unclear	No	Unclear	Moderate
NASEM, 2016 [10]	—	—	—	—	Yes	Unclear	Yes	Unclear	Yes	No	Moderate
NASEM, 2017 [4]	—	—	—	—	Yes	Yes	Yes	Yes	Yes	Yes	Good
Rowe, 2017 [26]	—	—	—	—	Yes	Yes	Yes	Unclear	No	Unclear	Moderate
Russell, 2016 [34]	Yes	Unclear	Yes	Yes	—	—	—	—	—	—	Good
Santesso, 2015 [29]	Yes	Yes	Unclear	Yes	—	—	—	—	—	—	Moderate
Snow, 2016 [35]	—	—	—	—	Yes	Yes	Yes	Yes	Yes	No	Good
Tunnecliff, 2015 [38]	Yes	Yes	Unclear	Yes	—	—	—	—	—	—	Good

^a^Cells are empty for publications where these particular criteria were not applicable.

^b^It was difficult to critically appraise primary research abstracts as key information may be excluded by authors for space considerations. Thus, the evaluation of overall quality should be interpreted with caution.

**Table 3:** Proposed typology of science communication strategies used by health scientists in the digital and social media ecosystem.

Type	Definition	Examples of each type of strategy
Content	Strategies to specify the type of health science–related content to be communicated	Announce new studies, research articles, and findings [10,21,23,24] and publish commentaries on health-related research [24]
Credibility	Strategies to support the credibility of health science–related content to be communicated	Present the confidence in the results (quality of evidence) on a scale [28,29] and disclose the sources of research funding [32]
Engagement	Strategies to increase public engagement with health science–related content to be communicated	Use hashtags [21,23,25] and update frequently [10,22]
Intention	Strategies to personalize health science–related content according to certain specific objectives or to convey a specific message	Make information actionable, that is, specify when to engage in an action and embed a trigger [32] and consider the usefulness of the research findings for the target audience [4,27,32]
Linguistics	Strategies to determine the linguistic microcomponents of the textual scientific information to be communicated	Minimize the use of, or replace, scientific jargon [27,28,31] and avoid acronyms [31]
Planification	Strategies to plan the operationalization of science communication, often in function of the audience(s) targeted	Develop a plan for engaging the targeted audience [10,33] and develop a YouTube channel devoted to disseminating research progress and findings along the way [33]
Presentation	Strategies to determine the structure and the visual presentation of the health science–related content to be communicated	Include pictures and, to a lesser extent, graphs [27] keep sentences and paragraphs short [31]
Social exchange	Strategies to increase and guide social exchanges related to the health science–related content	Encourage discussion, participation, and engagement [10,34,35] and converse with other users on topics related to health science on digital and social media [22]
Statistics	Strategies to determine the format of numeric and statistical scientific information	Present natural frequencies rather than percentages and probabilities [28] and be consistent in the numeric formats used [28]

After elimination of duplicates, 75 unique SC strategies distributed among the 9 types presented in Table 3 were identified in 15 of the publications reviewed (see Multimedia Appendix 2). No strategies were identified in 3 publications [26,30,38]. Only 13 strategies (13/75, 17%) were cited more than once: “Announcing new studies, research articles and findings” (*content*), “Use hashtags” (*engagement*), “Consider the usefulness of the research findings for the target audience” (*intention*), “Minimize the use of, or replace, scientific jargon” (*linguistics*), “Encourage discussion, participation and engagement on digital and social media” (*social exchange*), “Present the confidence in the results (quality of evidence) on a scale” (*credibility*), “Update frequently” (*engagement*), “Consider the interests of the target audience in the communication of research findings” (*intention*), “Arouse emotion” (*intention*), “Use a set of standard qualitative statements to express the magnitude of the effect” (*linguistics*), “Develop a plan for engaging the targeted audience” (*planification*), “Create and disseminate of summaries” (*content*), and “Use a question and answer layout” (*presentation*).

Interestingly, some SC strategies in the types *statistics* and *presentation* diverge. For instance, the strategies “Omit numbers” and “Use numbers instead of words when possible,” as well as “Omit tables” and “Use tables instead of narratives” appear contradictory.

Communication channels in the digital and social media ecosystem can be classified into 5 types underlining their primary purpose: (1) social networking platforms (ie, Twitter, Facebook, LinkedIn, Instagram, Google+, and Snapchat); (2) content-sharing platforms (ie, YouTube, Flickr, Scribd, and Slideshare); (3) digital research communities (ie, ResearchGate, Academia, and FigShare); (4) personal blogs and websites (ie, WordPress); (5) and social news aggregation and discussion platforms (ie, Reddit). The most frequently cited communication channels in reviewed publications were Twitter (n=13), blogs (n=9), Facebook (n=9), personal websites (n=6), YouTube (n=5), LinkedIn (n=3), Reddit (n=3), and Instagram (n=2).

## Discussion

### Summary of Evidence

This scoping review identified 18 publications that described a process of SC involving health scientists and the public operationalized in the digital and social media ecosystem. We identified 75 unique SC strategies and classified these into 9 types: content, credibility, engagement, intention, linguistics, planification, presentation, social exchange, and statistics. Moreover, we identified 5 types of communication channels in the digital and social media ecosystem: social networking platforms, content-sharing platforms, digital research communities, personal blogs and websites, and social news aggregation and discussion platforms.

**Figure 2:**
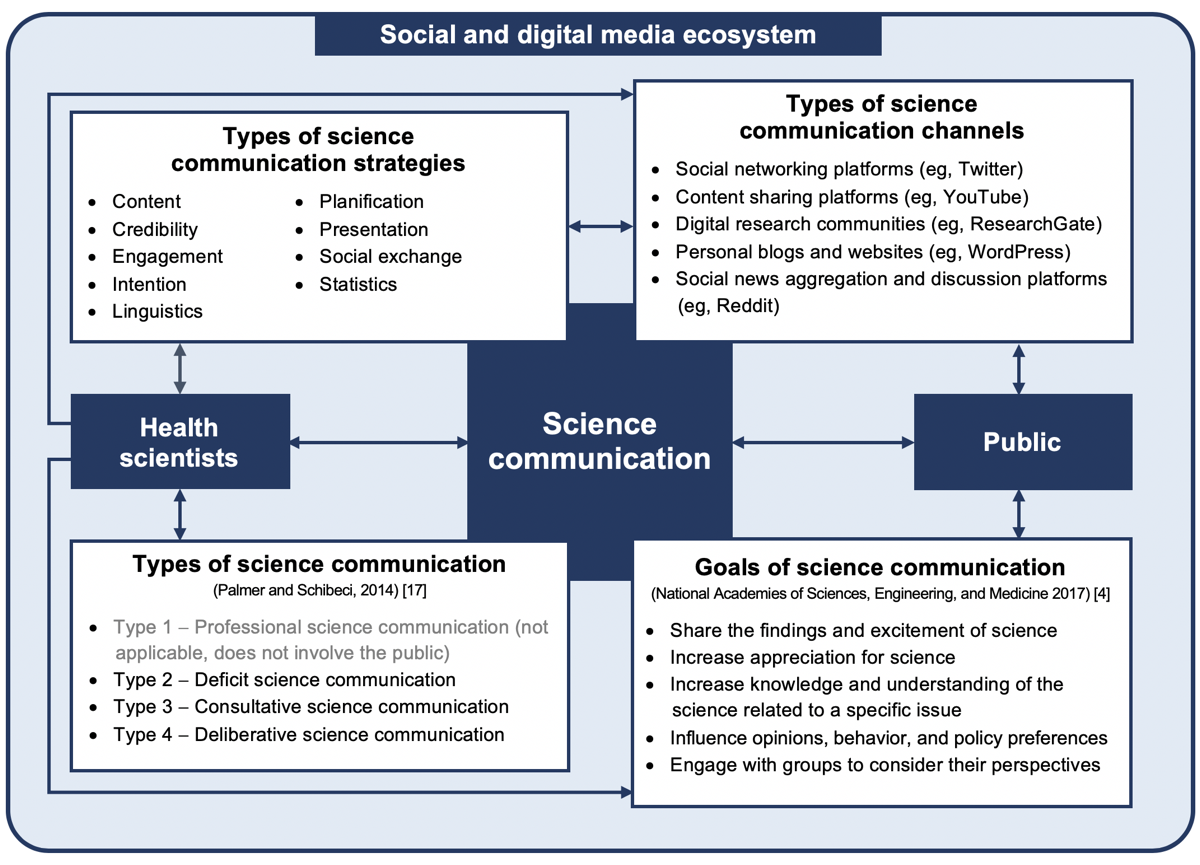
Schematization of the process of science communication between health scientists and the public in the digital and social media ecosystem.

To contextualize the results in relation with previous findings, we propose a schematization of the process of SC between health scientists and the public in the digital and social media ecosystem (see Figure 2). Health scientists, SC, and the public are the central concepts, and 4 elements are on the periphery: (1) the 9 types of SC strategies identified in this review; (2) the 5 types of communication channels in the digital and social media ecosystem identified in this review; (3) the 4 types of SC as described by Palmer and Schibeci [17]; and (4) the goals of SC as described by the National Academies of Sciences, Engineering, and Medicine [4].

According to reviewed literature, health scientists should aim to make scientific information useful for the public. This is a challenge, as the validity and usefulness of clinical studies has been previously debated. Indeed, critics point that health scientists often fail in either addressing an important health problem, generating new knowledge, or in producing rigorous research [39]. A total of 30% of health scientists think that less than half of the published literature in their field is reproducible, shedding light on the confidence scientists have in others’ findings [40]. Thus, this may impede scientists’ willingness to engage the public in SC. Employing SC strategies related to *planification*, *content*, *intention*, as identified in this review, could strengthen the process of SC. Indeed, by integrating SC in their research program and identifying prospectively the type of content to communicate and the objectives of SC, health scientists could improve the usefulness of scientific information for the public.

The expertise and trustworthiness of the person conveying a message have a strong effect on information credibility perceived by the general public [41]. In digital and social media, scientific information is often shared by the public and its validity is often questionable [23,42]. Diverging perceptions and opinions are fueled by appealing to ideologies or emotions rather than scientific facts. Indeed, reviewed literature suggests that scientific information that leads to amazement or fear in people is more likely to be shared than information that leads to sadness [4,32]. Asserting the *credibility* of the message by employing SC strategies underlining, for instance, the expertise of the scientist conveying the message and the confidence in the findings may help in counteracting messages that are not based on evidence.

In the context of SC, social exchanges between users must be encouraged to foster engagement with science but must also be framed by certain principles. We identified several SC strategies that may be used to increase *social exchanges* and foster the *engagement* of the public with health science. However, ethical principles to consider when using social media for SC were mentioned in only 1 reviewed article, in which authors referred more specifically to confidentiality and respect during online exchanges [34]. This is surprising considering that several reviews underline ethical issues surrounding the use of digital technology by health care professionals, such as boundary issues and potential conflicts of interests [4,43,44]. The extent to which these principles can be applied to health scientists has not been examined yet. Tensions between institutions’ social media policy and academic freedom could potentially discourage scientists from taking an active role in SC [45]. Further research should focus on identifying ethical principles regarding the use of digital and social media by health scientists.

Numeracy and health literacy are 2 concepts closely linked to SC that influence the public’s ability to properly evaluate health-related scientific information [46]. Contradictory findings were found with regard to SC strategies related to the *presentation* of information, *linguistics*, and the use of *statistics*. Although Glenton and Santesso [28] and Santesso and Rader [29] suggest the use of tables and the inclusion of numbers in SC, Barnfield and Pitts [27] and the National Academy of Sciences [32] suggest favoring a narrative format. More research is needed to identify the best strategies for facilitating the public’s understanding of scientific information through optimal presentation, linguistics, and statistics.

Engaging the public in the process of developing a SC plan is crucial to consider their needs and interests. In this review, 6 out of 18 publications mentioned including the public at some point in the development process of a SC plan. However, only 1 publication described how the public was involved in the process. In this study, members of the public participated in a focus group where they were invited to comment on lay summaries [27]. We expected the public to be more involved in the process of SC in reviewed studies. Indeed, several governmental organizations advocate for a conception of SC that is democratic and in which the principal actors have equal standing. Health scientists should strive for bidirectional communication by involving the targeted audience at the inception of a SC initiative.

### Strengths and Limitations

Strengths of this scoping review include the prospective publication of the protocol [12]. Moreover, the review was planned and conducted using a rigorous methodological framework [14] and was reported according to the PRISMA extension for scoping reviews to enhance replicability [15]. Although the scoping review methodology usually does not encompass quality assessment, we decided to include it to guide further research.

Limitations of this scoping review include the difficulty in synthesizing data from diverse sources of evidence. Few included sources of evidence were original research articles, and only 1 study employed an experimental design. Assessing publication quality proved difficult, considering the variability of publication types. Finally, differentiating the concept of SC from health literacy while performing the screening process proved to be a complex endeavor, the latter referring to individuals’ capacity to understand health information, and not science per se [35].

### Conclusions

Communicating findings of health research to the public is crucial to support self-care and to inform governmental decision making. Health scientists play a growing role in SC with the growth of digital and social media. This scoping review identified 75 SC strategies used by health scientists in the digital and social media ecosystem, which were categorized in 9 types. Results suggest health scientists currently use concurrently multiple SC strategies with a wide variety of purposes.

However, this scoping review identified that few empirical works have been conducted in this field. Further research should identify the barriers, facilitators, and ethical considerations inherent to the involvement of health scientists in the digital and social media ecosystem. Moreover, further research should focus on methods to increase public engagement with the health-related content shared (eg, through emotions) and developing and evaluating interventions to optimize the public’s understanding of complex notions related to science (ie, recognizing uncertainty, assessing the quality of evidence, qualifying the nature, and quantifying the strength of a relationship between 2 variables). Efforts should be undertaken to examine the appropriateness and effectiveness of the SC strategies used to improve public health–related outcomes. Conducting research in these areas may help to move beyond the deficit model of SC through the engagement of the public and consideration of its needs, interests, knowledge, and skills.

## Appendix

Multimedia Appendix 1 Search strategy for PubMed.

Multimedia Appendix 2 Science communication strategies identified in reviewed publications, classified by type.

## Abbreviations

PRISMA: Preferred Reporting Items for Systematic Reviews and Meta-Analyses
SC: science communication
