# Supplementary material for: Communicating Science in the Digital and Social Media Ecosystem: Scoping Review and Typology of Strategies Used by Health Scientists
Source: JMIR Public Health Surveill. 2019 Sep 3;5(3):e14447. doi: 10.2196/14447 (PMC6751098; doi:10.2196/14447)
Supplement: Multimedia Appendix 1 [file publichealth_v5i3e14447_app1.pdf]

Supplementary File 1. Search strategy for PubMed.

**PubMed (via NCBI)**	Search **(((((((((Researcher*[Title/Abstract]) OR Scientist*[Title/Abstract]) OR Expert*[Title/Abstract]) OR Investigator*[Title/Abstract])) AND (((Public[Title/Abstract]) OR Non-experts[Title/Abstract]) OR Population[Title/Abstract])) AND (((Health[Title/Abstract]) OR Biomedical[Title/Abstract]) OR MEdical[Title/Abstract])) AND (((((((Science*[Title/Abstract]) OR Research[Title/Abstract]) OR Scholarly[Title/Abstract]) OR Findings[Title/Abstract]) OR Results[Title/Abstract]) OR Innovation*[Title/Abstract]) OR Evidence[Title/Abstract])) AND ((((((Communicati*[Title/Abstract]) OR Disseminati*[Title/Abstract]) OR Populariz*[Title/Abstract]) OR Vulgariz*[Title/Abstract]) OR Diffusion[Title/Abstract]) OR Outreach[Title/Abstract])) AND (((((((((((((((((((((Disintermediation[Title/Abstract]) OR Internet[Title/Abstract]) OR Online[Title/Abstract]) OR Digital[Title/Abstract]) OR Web*[Title/Abstract]) OR Interactive[Title/Abstract]) OR Social media[Title/Abstract]) OR YouTube[Title/Abstract]) OR Twitter[Title/Abstract]) OR Facebook[Title/Abstract]) OR Reddit[Title/Abstract]) OR Instagram[Title/Abstract]) OR Vimeo[Title/Abstract]) OR LinkedIn[Title/Abstract]) OR Podcasts[Title/Abstract]) OR iTunes U[Title/Abstract]) OR Forum[Title/Abstract]) OR Discussion board[Title/Abstract]) OR Content sharing[Title/Abstract]) OR Video sharing[Title/Abstract]) OR Blog[Title/Abstract])** Filters: **Publication date from 2000/01/01 to 2018/12/31**	2018-03-04	**577**
