# Supplementary material for: Communicating Science in the Digital and Social Media Ecosystem: Scoping Review and Typology of Strategies Used by Health Scientists
Source: JMIR Public Health Surveill. 2019 Sep 3;5(3):e14447. doi: 10.2196/14447 (PMC6751098; doi:10.2196/14447)
Supplement: Multimedia Appendix 2 [file publichealth_v5i3e14447_app2.pdf]

Supplementary File 2. Science communication strategies identified in reviewed publications, classified by type.

Types	Science communication strategies	Archibald-2014	Barnfield-2017	Bin-2012	Bodison-2015	Breland-2017	Finch-2012	Glenton-2010	Lafferty-2015	Miranda-2014	NASEM-2013	NASEM-2016	NASEM-2017	Russel-2016	Santesso-2015	Snow-2016	Total
CONTENT	Share personal anecdotes related to health science.															1	1
CONTENT	Share news stories related to health science.															1	1
CONTENT	Publish commentaries on health-related research.						1										1
CONTENT	Upload conference research presentations in open online digital repositories.								1								1
CONTENT	Announcing new studies, research articles and findings.	1				1	1					1					4
CONTENT	Create a post related to health science on a content-sharing platform.											1					1
CONTENT	Share varied content.											1					1
CREDIBILITY	Disclose the sources of research funding.										1						1
CREDIBILITY	Present the confidence in the results (quality of evidence) on a scale.							1							1		2
CREDIBILITY	Emphasize that the experts who have formed the consensus have been right in the past.										1						1
CREDIBILITY	Communicate that scientists care about this issue and the methods used.										1						1
ENGAGEMENT	Announce blog posts on another social media platform.			1													1
ENGAGEMENT	Use hashtags.	1				1			1								3
ENGAGEMENT	Develop links between social media and traditional avenues of dissemination.	1															1
ENGAGEMENT	Use keywords and phrases that your audience might type in search engines.									1							1
ENGAGEMENT	Like news stories related to health science on social media platforms.															1	1
ENGAGEMENT	Update frequently.			1								1					2
ENGAGEMENT	Use the communication channel daily (Twitter).			1													1
INTENTION	Consider the interests of the target audience in the communication of research findings.		1								1						2
INTENTION	Consider the usefulness of the research findings for the target audience.		1								1		1				3
INTENTION	Create entertaining blog posts.			1													1
INTENTION	Create timely blog posts.			1													1
INTENTION	Communicate findings in a community-centered way.				1												1
INTENTION	Arouse emotion.										1		1				2
INTENTION	Make the messages memorable (simple, easy to visualize).										1						1
INTENTION	Make the messages actionable (specify when to act, and embed a trigger to take action).										1						1
INTENTION	Make the messages motivational and plausible.										1						1
INTENTION	Make the messages positive and reassuring.										1						1
INTENTION	Make the messages clear.										1						1

INTENTION	Show personality in the messages.											1					1
LINGUISTICS	Minimize the use of, or replace, scientific jargon.		1					1		1							3
LINGUISTICS	Remove most definitions and consider employing a glossary of terms.		1														1
LINGUISTICS	Use a set of standard qualitative statements to express the magnitude of the effect and our confidence in these results.							1							1		2
LINGUISTICS	Use the active voice.									1							1
LINGUISTICS	Use catchy titles.									1							1
LINGUISTICS	Avoid acronyms.									1							1
LINGUISTICS	Make sure the flow of information follows principles of linguistics frameworks.														1		1
PLANIFICATION	Develop a plan for engaging the targeted audience.				1							1					2
PLANIFICATION	Listen to the needs of targeted audience.											1					1
PLANIFICATION	Develop a YouTube channel expressly devoted to disseminating research progress and findings along the way.				1												1
PLANIFICATION	Develop a website expressly devoted to disseminating research progress and findings along the way.				1												1
PLANIFICATION	Consider the perceptions of social norms that can shape audiences' behaviors and attitudes.												1				1
PLANIFICATION	Consider the socioeconomic knowledge gap in planning how to engage segments of the public not already interested in science.												1				1
PLANIFICATION	Tailor health science communication to the preferences of different audiences.												1				1
PRESENTATION	Include pictures and, to a lesser extent, graphs.		1														1
PRESENTATION	Use multimedia and visuals.									1							1
PRESENTATION	State what is known and what is unknown.										1						1
PRESENTATION	Create and disseminate of summaries.		1					1									2
PRESENTATION	Use a question and answer layout.		1												1		2
PRESENTATION	Omit tables (Contradictory, B1, D1).		1														1
PRESENTATION	Include of a "quick read" section.		1														1
PRESENTATION	State the aim of science communication early.		1														1
PRESENTATION	Create blog posts of about 700 words.			1													1
PRESENTATION	Use tables instead of narratives (Contradictory, B2, C1).							1									1
PRESENTATION	Keep sentences and paragraphs short.									1							1
PRESENTATION	Consider different forms of structure while designing messages.										1						1
PRESENTATION	Use narratives (Contradictory, C2).										1						1
PRESENTATION	Provide a quantitative description of effects in table format (Contradictory, D2).														1		1
SOCIAL EXCHANGE	Develop rules of engagement in the community.													1			1
SOCIAL EXCHANGE	Ensure privacy.													1			1
SOCIAL EXCHANGE	Encourage discussion, participation and engagement on digital and social media.											1		1		1	3
SOCIAL EXCHANGE	Converse with other users on topics related to health science on digital and social media.			1													1

SOCIAL EXCHANGE	Encourage interactions and collaboration amongst researchers, practitioners and general public on digital and social media.						1										1
SOCIAL EXCHANGE	Make connections between active members of a community group on digital and social media.													1			1
SOCIAL EXCHANGE	Establish a sense of community by acknowledging the contributions of members on digital and social media.													1			1
SOCIAL EXCHANGE	Monitor the community on digital and social media.													1			1
SOCIAL EXCHANGE	Invite members to introduce themselves on digital and social media.													1			1
SOCIAL EXCHANGE	Introduce icebreakers on digital and social media.													1			1
SOCIAL EXCHANGE	Comment on blogs.															1	1
STATISTICS	Omit numbers (Contradictory, A1).		1														1
STATISTICS	Use numbers instead of words when possible (Contradictory, A2).							1									1
STATISTICS	Present absolute rather than relative risk formats.							1									1
STATISTICS	Present natural frequencies rather than percentages and probabilities.							1									1
STATISTICS	Be consistent in the numeric formats used.							1									1

* Contradictory strategies are grouped in letter pairs (e.g., A1 and A2, B1 and B2).
